# Psychological wellbeing of Australian community health service staff during the COVID-19 pandemic: a longitudinal cohort study

**DOI:** 10.1186/s12913-023-09382-y

**Published:** 2023-04-26

**Authors:** Sara Holton, Karen Wynter, Anna Peeters, Alexandra Georgalas, Ann Yeomanson, Bodil Rasmussen

**Affiliations:** 1 School of Nursing and Midwifery, Faculty of Health, Deakin University, Geelong, VIC 3220 Australia; 2 The Centre for Quality and Patient Safety Research in the Institute of Health Transformation, Deakin University - Western Health Partnership, St Albans, VIC 3021 Australia; 3 Institute of Health Transformation, Faculty of Health, Deakin University, Geelong, VIC 3220 Australia; 4 Victorian Healthcare Association, Melbourne, VIC 3000 Australia; 5 Faculty of Health Sciences, University of Southern Denmark and Steno Diabetes Center, Copenhagen, Denmark; 6 Faculty of Health and Medical Sciences, University of Copenhagen, Copenhagen, Denmark

**Keywords:** COVID-19, Australia, Community health services, Psychosocial, Longitudinal study

## Abstract

**Background:**

Hospital clinical staff have reported poor psychosocial wellbeing during the COVID-19 pandemic. Little is known about community health service staff who undertake various roles including education, advocacy and clinical services, and work with a range of clients. Few studies have collected longitudinal data. The aim of this study was to assess the psychological wellbeing of community health service staff in Australia during the COVID-19 pandemic at two time points in 2021.

**Methods:**

A prospective cohort design with an anonymous cross-sectional online survey administered at two time points (March/April 2021; n = 681 and September/October 2021; n = 479). Staff (clinical and non-clinical roles) were recruited from eight community health services in Victoria, Australia. Psychological wellbeing was assessed using the Depression, Anxiety and Stress Scale (DASS-21) and resilience using the Brief Resilience Scale (BRS). General linear models were used to measure the effects of survey time point, professional role and geographic location on DASS-21 subscale scores, adjusting for selected sociodemographic and health characteristics.

**Results:**

There were no significant differences in respondent sociodemographic characteristics between the two surveys. Staff’s mental health declined as the pandemic continued. Adjusting for dependent children, professional role, general health status, geographic location, COVID-19 contact status and country of birth; depression, anxiety and stress scores were significantly higher for respondents in the second survey than the first (all p < 0.001). Professional role and geographic location were not statistically significantly associated with scores on any of the DASS-21 subscales. Higher levels of depression, anxiety and stress were reported among respondents who were younger, and had less resilience or poorer general health.

**Conclusions:**

The psychological wellbeing of community health staff was significantly worse at the time of the second survey than the first. The findings indicate that the COVID-19 pandemic has had an ongoing and cumulative negative impact on staff wellbeing. Staff would benefit from continued wellbeing support.

**Supplementary Information:**

The online version contains supplementary material available at 10.1186/s12913-023-09382-y.

## Background

Healthcare workers have experienced considerable emotional distress during the COVID-19 pandemic, particularly nurses and clinical staff who have had direct contact with patients diagnosed with COVID‐19 [1–8]. Most studies to date have focused on hospital clinical staff providing acute care [1], few studies have investigated the impact of the pandemic on staff, both in clinical and non-clinical roles, who work in primary health care or community health services. Community health service staff tend to be a more heterogeneous group than hospital clinical staff and therefore, it may not be possible to generalise the findings of studies about hospital clinical staff to those staff working in community settings. Community health service staff may also have faced different challenges during the pandemic such as leading public health COVID-19 prevention strategies, providing care in client’s homes or providing services via telehealth [1, 9, 10].

Community health services in Australia provide publicly funded primary healthcare which focuses on people with, or at risk of, poorer health. They deliver health care in partnership with general practice, privately funded health services and other health and support services, and provide a range of services including allied health services, child health services, chronic disease management (including support for self-management), dental health services, disability services, drug and alcohol services, family planning, health promotion, home and community care services, medical services, mental health services, post-acute care services and refugee health [11].

A recent review about the impact of COVID-19 on the mental health of healthcare workers found no studies which related to community health service staff [1]. A few studies which have been conducted in a primary or community health setting have focused on particular groups of healthcare workers such as nurses or personal care assistants [9, 12, 13]; been restricted to particular countries including the Philippines [12], the United Kingdom (UK) [14] and New Zealand [9] or settings such as residential care homes or domiciliary care [14]; or have been small qualitative studies [9, 14]. None have investigated the experiences and perspectives of community health service staff undertaking a range of roles (including both clinical and non-clinical) in services based in different geographical locations. Few have investigated their psychological wellbeing including symptoms of depression, anxiety and stress, and resilience despite evidence demonstrating that many healthcare workers have experienced clinically significant levels of psychological distress during the COVID-19 pandemic [4, 8, 15–17]. Studies conducted during the pandemic have also shown an association between anxiety, stress and resilience among health care workers [18, 19], and suggest that although individual levels of resilience have not changed during the pandemic [20–22], resilience may be protective against psychological distress [22].

Little is known about the psychosocial effects of the COVID-19 pandemic on community health service staff in Australia which has experienced relatively fewer COVID-19 deaths per million people compared to other countries [23]. Community health service staff in Australia undertake various primary healthcare roles including education, advocacy and clinical services; and work with a range of communities including those which experience disadvantage and are culturally and linguistically diverse, many of which have been disproportionally affected by COVID-19. During the COVID-19 pandemic community health services in Australia adapted many of their services to non-face-to-face delivery including telehealth as well as providing COVID‐19 services such as respiratory clinics, testing sites, care for positive patients in community settings, and working with people in high‐risk accommodation settings [10].

The aim of this study was to assess the psychological wellbeing of community health service staff in Australia during the COVID-19 pandemic in 2021. The specific objectives of the study were to investigate: (1) the level of emotional distress (symptoms of depression, anxiety and stress) experienced by community health service staff at two time points; and (2) the impact of the time point (different time points during the pandemic), professional role (clinical vs. non-clinical) and geographic location (metropolitan vs. regional/rural) on staff’s levels of depression, anxiety and stress.

## Methods

### Study design, setting and participants

The study compared data collected from two independent groups via a cross-sectional survey administered at two time points: (1) after the second ‘wave’ of the COVID-19 pandemic in Victoria, Australia (March/April 2021); and (2) six months later (September/October 2021).

Surveys were distributed at two time points to enable assessment of the ‘immediate’ and ‘longer-term’ impact of the pandemic on the psychological wellbeing of community health service staff. The six-month interval between surveys was the result of pragmatic considerations including the lack of certainty at the commencement of the study about how often and when the state of Victoria would experience future COVID-19 ‘waves’ and their severity, and the time-sensitive nature of the research. The collection of data at only two time points was chosen to minimise the burden on community health staff so that they could continue to provide care and services to their clients with minimal interruption.

At the time of the first survey, the total number of confirmed COVID-19 cases in Victoria since the beginning of the pandemic was just over 20,000 and over 47,000 COVID-19 vaccination doses had been administered [24]. Restrictions which had been implemented by the state government to prevent and slow the spread of infection during the ‘second wave’ of the COVID-19 pandemic were easing and workers could return to onsite work, visitors were allowed in private homes, and masks were required only on public transport and in hospitals [25]. Melbourne, the capital city of Victoria, had experienced over 200 days of ‘lockdown’ at the time of the second survey and the total number of COVID-19 cases had increased to over 27,000. Restrictions were implemented in August 2021 for both metropolitan and regional Victoria and partially lifted at the end of September 2021 in order to manage the ‘third wave’ (Delta) [26].

Community health service staff were recruited from eight community health services based in metropolitan and regional/rural locations in the state of Victoria, Australia.

### Procedure

At each survey time point, the CEO/Executive team of each participating community health service sent an email invitation to their staff via their organisation’s group email addresses. The email explained the project and included a link to the online survey (Supplementary Material). A participant information sheet was also attached. A reminder email was sent two weeks after the initial invitation email. The first survey was open for seven weeks (22 March 2021–7 May 2021), and the second for eight weeks (7 September 2021–2 November 2021). Completion of the survey was taken as informed consent.

The surveys were available in Qualtrics (an online survey platform) and took approximately 10–15 minutes to complete. The surveys were based on those used in similar study conducted by members of the research team with hospital clinical staff in Australia and Denmark [3–5, 8]. The surveys used mostly fixed-response questions and assessed respondents’:

1. Sociodemographic and employment characteristics (e.g. age, highest level of education, professional role (clinical vs. non-clinical), number of years practiced, number of years employed at their community health service, work location (metropolitan vs. regional/rural), employment status – full/part time, casual/bank etc.);
2. Current health status (i.e. overall self-reported general health status).
3. Psychological wellbeing (e.g. symptoms of depression, anxiety and stress (Depression, Anxiety and Stress Scale, DASS-21); and resilience (Brief Resilience Scale, BRS)).

The surveys were the same at each time point.

Both surveys were anonymous but to enable longitudinal matching, respondents to each survey were asked to create a unique identification code using a specific combination of letters and numbers from their personal details (e.g. name and date of birth). The same instructions for generating this code were included in both surveys as well as examples.

#### Measures

General health status: assessed using a single question: ‘In general, would you say your health is …? Very poor, poor, fair, good or excellent’. For the analyses these were categorised as ‘very poor/poor/fair’ or ‘excellent/good’.

DASS-21: Depression, anxiety and stress symptoms during the past week were assessed using the Depression, Anxiety and Stress Scale (DASS-21) [27]. Scores on each subscale range from 0 (no distress) to 21 (most distressed). Clinical cut-off points have been established for depression (mild, 5–6; moderate, 7–10; severe, 11–13; extremely severe, ≥ 14), anxiety (mild, 4–5; moderate, 6–7; severe, 8–9; extremely severe, ≥ 10) and stress (mild, 8–9; moderate, 10–12; severe, 13–16; extremely severe, ≥ 17) [27].

The Brief Resilience Scale (BRS) [28]: was used to assess how staff were coping with the challenges of the pandemic. Higher scores indicate greater resilience.

### Data management and analysis

Quantitative data were analysed using IBM SPSS Statistics version 26 (IBM Corp., Armonk, NY, USA). Data were summarised using descriptive statistics.

In order to compare responses from surveys 1 and 2, data were first matched using the unique identification codes generated by the respondents. Only 92 paired responses from respondents who completed both surveys and responded to both requests to generate the code were identified. Therefore, the data from the two surveys were treated as independent samples.

Chi-square or Mann-Whitney U tests were used as appropriate to test for differences between the two surveys (time points) in terms of sociodemographic characteristics and psychological wellbeing.

DASS-21 subscale scores and proportion scoring in clinical ranges were calculated as outlined by the instrument’s authors [27]. This assisted in determining the proportion of community health workers who have experienced ‘normal’, ‘mild’, ‘moderate’, ‘severe’ or ‘extremely severe’ depression, anxiety or stress. These ‘labels’ assist in characterising the degree of distress severity relative to the general population. Using one-sample t-tests, the study’s findings were compared to normative data for the DASS-21 (available in the DASS manual) as well as prevalence rates of emotional distress in the general population, DASS-21 scores reported in peer-reviewed journal articles (eg Crawford et al 2011 [29]) including those of hospital clinical staff during the COVID-19 pandemic (e.g. Holton et al. [4]). In accordance with the DASS-21 guidelines, one missing item was allowed for each subscale. Cases with two or more missing values for each subscale were removed.

BRS scores were calculated as outlined by the instrument’s authors [28].

DASS-21 subscale and BRS scores were compared between the two surveys (time points) using independent samples t-tests (if distributions were normal) or Mann-Whitney U tests (if non-normal). Associations between subscale scores and demographic, health and COVID-19 responses were tested using appropriate tests (Chi-square or Mann-Whitney U tests).

The effects of survey time point, professional role and geographic location on DASS-21 subscale scores were simultaneously tested using general linear models (GLM), controlling for demographic and health variables which were significantly associated (p < 0.05) with DASS subscale scores in univariate analyses.

## Results

### Sample and response

Approximately 3,176 staff were employed at the eight participating health services at the commencement of the study and 681 (approximately 21.4%) completed the first survey and 479 (15.1%) completed the second survey.

Most respondents in both surveys identified as women and were born in Australia; fewer than half lived with dependent children and of these, most had school-aged children (Table 1).

**Table 1:** Respondents’ sociodemographic and employment characteristics (Surveys 1 & 2)

Characteristic	Survey 1 N (%)	Survey 2 N (%)	p value
Gender identity	n = 646	n = 456	p = 0.295
Woman	523 (81.0%)	380 (83.3%)	
Man	112 (17.3%)	65 (14.3%)	
Prefer not to disclose/self-described	11 (1.7%)	11 (2.4%)	
Age	n = 637	n = 443	p = 0.714
Range (years)	19–76	21–71	
Mean (SD)	44.2 (11.7)	44.4 (12.3)	
Country of birth	n = 638	n = 456	p = 0.764
Australia	475 (74.5%)	344 (75.4%)	
Overseas	163 (25.5%)	112 (24.6%)	
Live with dependent children	n = 643	n = 454	p = 0.448
Yes	268 (41.7%)	178 (39.2%)	
No	375 (58.3%)	276 (60.8%)	
Dependent children attend			
Child care	51 (7.5%)	37 (7.7%)	
Primary school	136 (20.0%)	92 (19.2%)	
Secondary school	144 (21.1%)	74 (15.4%)	
Employment status	n = 643	n = 456	p = 0.474
Permanent full-time	205 (31.9%)	143 (31.4%)	
Permanent part-time	257 (40.0%)	195 (42.8%)	
Fixed-term full-time	84 (13.1%)	51 (11.2%)	
Fixed-term part-time	72 (11.2%)	51 (11.2%)	
Other (casual, ‘mixture’)	5 (0.8%)	16 (3.5%)	
Geographic location	n = 634	n = 449	p = 0.320
Metropolitan	456 (71.9%)	336 (74.8%)	
Regional/rural	178 (28.1%)	113 (25.2%)	
Professional role	n = 522	n = 382	p = 0.980
Clinical	299 (57.3%)	220 (57.6%)	
Non-clinical	223 (42.7%)	162 (42.4%)	
Manager of one or more employees	n = 639	n = 452	p = 0.373
Yes	126 (19.7%)	100 (22.1%)	
No	513 (80.3%)	352 (77.9%)	
Years worked in the community health sector	n = 635	n = 450	p = 0.815
Range (years)	0–45	0–44	
Mean (SD)	9.6 (8.3)	9.5 (8.7)	
Years employed at current health service	n = 631	n = 449	p = 0.634
Range (years)	0–37	0–40	
Mean (SD)	6.3	6.1 (6.7)	

Most respondents were employed on a permanent basis; just over half were part-time (employed on either a permanent or fixed-term basis) and the remainder were full-time (employed on either a permanent or fixed-term basis). More than half of the respondents were health professionals (clinical role) and approximately one in five was a manager of one or more employees. On average respondents had worked in the community health services sector for over 9 years and at their current community health service for over 6 years. Almost three-quarters of the respondents worked in metropolitan Melbourne. There were no statistically significant differences in the demographic or employment characteristics of the samples at each survey time point (Table 1).

No respondents had been diagnosed with COVID-19 at either survey time point. Although the majority of respondents had had no contact with people diagnosed with COVID-19, a significantly greater proportion of survey 2 respondents had had contact than those in survey 1. About one in ten survey 1 respondents and about one in five survey 2 respondents had been in direct contact with people (either at work or outside of work) with a COVID-19 diagnosis and had experienced associated self-isolation and testing (with negative results) (Table 2).

**Table 2:** Respondents’ COVID-19 and health status (Surveys 1 & 2)

	Survey 1 N (%)	Survey 2 N (%)	p value
COVID-19 contact status	n = 582	n = 423	
No direct contact with people with known COVID 19 diagnosis	510 (87.6%)	337 (79.7%)	**p < 0.001**
Direct contact with people who have had COVID 19 diagnosis which resulted in self-isolation or testing (with a negative COVID 19 result)	72 (12.4%)	86 (20.3%)	
Diagnosed with COVID 19	0 (0%)	0 (0%)	
General health status	n = 614	n = 438	**p = 0.004**
Excellent/good	489 (79.6%)	314 (71.7%)	
Fair/poor/very poor	125 (20.4%)	124 (28.3%)	

### General health status

Most respondents in both surveys rated their general health status as excellent/good. However, survey 2 respondents were significantly less likely to rate their health as excellent/good than those in survey 1 (Table 2).

### Psychological wellbeing

There was no significant difference in respondents’ mean scores on the Brief Resilience Scale at each of the survey time points (Survey 1: 3.48 vs. Survey 2: 3.43; p = 0.363).

A considerable proportion of the community health services staff surveyed reported moderate to extremely severe symptoms of depression (15.3%), anxiety (9.6%) or stress (12.7%) at the time of the first survey. However, the proportion increased as the pandemic progressed with almost a third (31.3%) of survey 2 respondents reporting moderate to extremely severe symptoms of depression, 19.1% reporting symptoms of anxiety and a quarter (25.6%) stress (Table 3).

**Table 3:** Respondents’ psychological wellbeing (DASS-21)

Scale		Survey 1	Survey 2	Sig	Ranges for clinical cut-off points	Survey 1	Survey 2
DASS-21 Depression (range 0–21)	Mean (SD)	*n = 610*	*n = 434*		Normal (0–4)	452 (76.2%)	231 (54.7%)
		3.11 (3.69)	4.91 (4.14)	**p < 0.001**	Mild (5–6)	50 (8.4%)	59 (14.0%)
					Moderate (7–10)	56 (9.4%)	88 (20.9%)
					Severe (11–13)	22 (3.7%)	24 (5.7%)
					Extremely Severe (14+)	13 (2.2%)	20 (4.7%)
DASS-21 Anxiety (range 0–21)	Mean (SD)	*n = 610*	*n = 435*		Normal (0–3)	480 (79.3%)	299 (69.7%)
		2.08 (2.66)	2.96 (3.22)	**p < 0.001**	Mild (4–5)	67 (11.1%)	48 (11.2%)
					Moderate (6–7)	24 (4.0%)	35 (8.2%)
					Severe (8–9)	20 (3.3%)	22 (5.1%)
					Extremely Severe (10+)	14 (2.3%)	25 (5.8%)
DASS-21 Stress (range 0–21)	Mean (SD)	*n = 608*	*n = 436*		Normal (0–3)	456 (76.4%)	261 (61.3%)
		4.96 (3.99)	6.71 (4.47)	**p < 0.001**	Mild (4–5)	65 (10.9%)	56 (13.1%)
					Moderate (6–7)	47 (7.9%)	58 (13.6%)
					Severe (8–9)	24 (4.0%)	40 (9.4%)
					Extremely Severe (10+)	5 (0.8%)	11 (2.6%)

Cronbach’s alphas for the DASS-21 subscale scores at each time point were: 0.896/0.887 for depression, for 0.757/0.807 for anxiety and 0.884/0.886 for stress.

There was a significant difference between the DASS-21 subscale mean scores at surveys 1 and 2. Mean scores on the depression, anxiety and stress subscales were significantly higher for respondents in the second survey than the first (Table 3).

In the general linear models, the main effect for time point was significant for all three subscales. Compared with the first time point, the second time point was associated with significantly higher depression, anxiety and stress scores (all p < 0.001). Professional role and geographic location were not statistically significant associated with scores on any of the three subscales. Lower levels of depression, anxiety and stress were reported by respondents who were older and those who had more resilience. Poorer general health and not living with dependent children were significantly associated with higher levels of depression (Table 4).

**Table 4:** Impact of time point, professional role and geographic location on respondents’ depression, anxiety and stress scores

Variable	Depression					Anxiety					Stress				
	B	Sig.	95% CI	F(1, 792)	Partial η2	B	Sig.	95% CI	F(1, 951)	Partial η2	B	Sig.	95% CI	F(1, 951)	Partial η2
Live with dependent children															
No	0.691	**0.007**	0.190,1.192	7.323	0.009	0.315	0.104	− 0.065,0.695	2.643	0.003	0.307	0.268	− 0.236,0.850	1.229	0.002
Yes (ref)															
Age	− 0.031	**0.004**	− 0.052,-0.010	8.299	0.010	− 0.042	**< 0.001**	− 0.058,-0.026	26.507	0.033	− 0.050	**< 0.001**	− 0.073,-0.027	18.007	0.022
BRS Mean score	-1.765	**< 0.001**	-2.115,-1.415	97.851	0.111	-1.066	**< 0.001**	-1.332,-0.801	62.028	0.073	-1.922	**< 0.001**	-2.301,-1.542	98.957	0.112
Professional role															
Non-clinical	0.098	0.704	− 0.407,0.602	0.144	0.000	0.127	0.515	− 0.256,0.509	0.423	0.001	0.198	0.478	− 0.349,0.744	0.505	0.001
Clinical (ref)															
Self-rated general health															
Fair/poor	2.008	**< 0.001**	1.397,2.618	41.674	0.050	1.383	**< 0.001**	0.920,1.846	34.437	0.042	1.359	**< 0.001**	0.697,2.021	16.217	0.020
Good/excellent (ref)															
Location															
Regional/rural	− 0.068	0.815	− 0.641,0.504	0.055	0.000	− 0.041	0.853	− 0.475,0.393	0.034	0.000	− 0.566	0.074	-1.187,0.055	3.203	0.004
Metro (ref)															
COVID-19 contact status															
Contact	0.473	0.165	0.194, 1.139,	1.935	0.002	0.026	0.920	− 0.481, 0.533	0.010	0.000	0.399	0.278	− 0.322, 1.121	1.180	0.002
No contact (ref)															
Country of birth															
Australia	0.252	0.409	− 0.348, 0.853	0.681	0.001	− 0.120	0.606	− 0.576, 0.336	0.266	0.000	0.704	**0.034**	0.054, 1.354	4.517	0.006
Other (ref)															
Survey time point															
Two	1.688	**< 0.001**	1.191,2.186	44.324	0.054	0.881	**< 0.001**	0.503, 1.258	20.969	0.026	1.811	**< 0.001**	1.272, 2.351	43.480	0.053
One (ref)															

B - Unstandardised coefficient, CI - confidence intervals, η2 eta squared, ref – reference category

## Discussion

This study assessed the psychological wellbeing of community health service staff in Australia at two time points during the COVID-19 pandemic in 2021 and found that staff’s wellbeing declined as the pandemic continued. The wellbeing of staff was significantly worse at the second study time point compared to the first. These findings are similar to those of the few other longitudinal studies in this field which have also found that the wellbeing of healthcare workers in clinical settings became worse during subsequent waves of the pandemic [8, 30]. It is likely that concerns about being infected with COVID-19, family’s health, redeployment, accessing and using personal protective equipment, and changes in the delivery of services to clients and stakeholders have had a continued and cumulative effect on staff wellbeing and resulted in poorer psychological wellbeing as the pandemic progressed [8, 30].

Few studies have compared the psychological impact of the COVID-19 pandemic on health service staff in different professional roles particularly those in clinical and non-clinical roles; most studies to date have focused on ‘front-line’ clinical staff [1]. No significant difference was found between the psychological wellbeing of respondents in a clinical role compared to those in a non-clinical role at either time point in this study. Nevertheless, previous studies indicate mixed findings about the association between professional role and psychological distress during the COVID-19 pandemic. The findings of some studies suggest that being in a clinical role is associated with poorer wellbeing than being in a non-clinical role [30, 31]. Yet others have found that both clinical and non-clinical staff have experienced poor wellbeing during the pandemic [32]. It maybe that although clinical staff are more likely to have had contact with COVID-19 patients and experienced increased clinical demands, fear of infection and prolonged periods of wearing PPE; non-clinical staff may have experienced anxiety and stress about potential exposure to COVID-19 from working in a health service setting as well as a greater sense of contribution and inclusion in the workforce and accordingly greater physical and emotional exhaustion [31, 32].

Little is known about the association between geographic location and healthcare worker wellbeing during the COVID-19 pandemic. A rapid review about the impact of COVID-19 on the mental health of healthcare workers [1] identified only one study [33] which had assessed the association between location and psychological wellbeing with the results indicating that living in a rural area was an independent factor for anxiety among medical healthcare workers. In contrast, the current study found no significant difference between the psychological wellbeing of community health service staff working in a metropolitan location and those in a regional or rural area at either study time point. This may be the result of the similar COVID-19 restrictions including ‘lockdowns’ which were implemented by the Victorian state government during the study time period for both metropolitan Melbourne and regional Victoria in an attempt to reduce community transmission and the number of COVID-19 cases [34].

Similar to the findings of others [1, 6, 35], respondents in this study who were younger reported higher levels of depression, anxiety and stress. Age is likely to be correlated with years of professional experience and health service staff with greater years of experience may feel more confident to undertake their role due to their knowledge, expertise and prior experience and this may be protective of their wellbeing [3, 36, 37].

Poorer general health status was found in this study to be associated with higher levels of depression, anxiety and stress. This is consistent with the results of recent rapid and meta reviews about the impact of the COVID-19 pandemic on healthcare workers which found that a pre-existing illness was associated with poorer psychological wellbeing [1, 6]. People with a chronic illness may be at greater risk of COVID-19 infection and hospitalisation and accordingly, due to their vulnerability, experience greater psychological distress [38]. Poor health status has been shown to be a significant risk factor for the development of mental health problems including depression [39]. Therefore, the greater levels of psychological distress experienced by the respondents in this study who reported poorer general health status may be the result of mediating factors such as symptom burden, chronic pain, and reduced quality of life [40] as well as the impacts of the COVID-19 pandemic.

As in other studies [1, 35], resilience was found in this study to be protective of psychological wellbeing. It is likely that health service staff with greater levels of resilience were able to cope more effectively with the stressful situation of the COVID-19 pandemic [41].

Similar to other studies investigating factors associated with healthcare worker wellbeing during the COVID-19 pandemic [3, 42], this study found that having school-aged children was associated with better psychological wellbeing. As previously suggested, living with other people appears to be protective of mental health [42, 43].

### Strengths and limitations

This is one of the first studies about the impact of the COVID-19 pandemic on community health service staff’s psychological wellbeing in Australia conducted at more than one time point. The study design enabled data to be collected about changes in psychological wellbeing as the pandemic progressed. A major strength of the study is the inclusion of both clinical and non-clinical staff from a range of community health services located in different geographical areas. Validated psychometric instruments were used to assess depression, anxiety, stress and resilience. In order to minimise the burden on community health service staff, it was possible to collect data at only two time points. Future studies should collect longitudinal data both during subsequent waves of the pandemic and after the pandemic so that the long-term psychosocial effects on community health staff can be ascertained. Due to the small number of respondents who generated a unique identification code and completed both surveys, responses were not matched across both surveys. As a result, the sample is different at each survey time point but no significant differences in respondents’ sociodemographic characteristics were identified.

### Implications for community health service policy and practice

Similar to the recommendations and findings of others [9, 14, 44], the findings of this study indicate that community health service staff would benefit from additional and ongoing organisational initiatives to support their psychological wellbeing,

## Conclusions

The psychological wellbeing of community health service staff in Australia deteriorated as the COVID-19 pandemic continued. Staff would benefit from continued and ongoing wellbeing initiatives and support.

## Electronic supplementary material

Below is the link to the electronic supplementary material.

Supplementary Material 1

Supplementary Material 2

## Data Availability

The datasets used and/or analysed during the current study are available from the corresponding author on reasonable request.
