# Supplementary material for: Psychological wellbeing of Australian community health service staff during the COVID-19 pandemic: a longitudinal cohort study
Source: BMC Health Serv Res. 2023 Apr 26;23:405. doi: 10.1186/s12913-023-09382-y (PMC10131448; doi:10.1186/s12913-023-09382-y)
Supplement: Supplementary file 1 — Supplementary Material 1 [file 12913_2023_9382_MOESM1_ESM.pdf]

Introduction

Psychosocial impact of COVID-19 on community health service staff

Survey 1

Thank you for your interest in the research study, “Psychosocial impact of COVID-19 on community health service staff”. This study has been approved by the Deakin University Human Research Ethics Committee.

The participant information sheet tells you more about the study and can be accessed via this link: [INSERT LINK TO PICF]

Please read through the participant information sheet.

We will not ask you to write your name on this survey but will ask you to create an unique code number known only to you. We will ask you to complete another follow-up survey in about six months and the personal code you generate will then be used to link your responses to this survey (February/March 2021) and the second one (August/September 2021) if you choose to participate in both.

There are no right or wrong answers to the questions in this survey. We are simply interested in your experiences, thoughts and opinions. If you are unsure about how to answer a question, please mark the response which corresponds most closely to how you feel.

The survey will take approximately 10 - 15 minutes to complete.

Your completion of the survey indicates your consent to participate in the study.

Thank you for your participation. If you have any questions about the study please contact Dr Sara Holton at email: s.holton@deakin.edu.au.

Please create your own unique code (no spaces, no commas, no full stops) as described below:

The first letter of your name (lower case)
Your month of birth (use two numbers)
The first two letters of your parent/guardian's surname (lower case)

For example, the codes for the following people would be:
Susan Jones is born in February and their parent's surname is Brown.
s02br
Lei Zhang is born in June and their parent's surname is Zhang.
l06zh

The format of this code is required so that you can remember it if you decide to participate in the second survey (August/September 2021).

Demographics

Section 1. Some questions about you

What is your gender identity?

- ○ Woman

- ◯ Man
- ◯ Prefer not to disclose
- ◯ Self-described

What was your age at your last birthday?

In which country were you born?

Do you live with dependent children?

- ◯ Yes
- ◯ No

If yes, do your dependent children attend ...?

- ☐ Child care
- ☐ Primary school
- ☐ Secondary school

What is your employment status at your organisation?

- ◯ Permanent full-time
- ◯ Fixed-term full-time
- ◯ Permanent part-time
- ◯ Fixed-term part-time
- ◯ Casual / bank / pool
- ◯ Other (please specify)

Which of the following categories best describes your current position?
(Please select one option only)

- Nursing employees (e.g. nurse educator, midwifery & nursing professionals, enrolled and mothercraft nurses)
- Medical employees (e.g. medical practitioners, VMOs, interns)
- Allied health professionals (e.g. physiotherapists, occupational therapists)
- Other health professionals (e.g. psychologists, pharmacists, radiographers, medical scientists, medical technicians, radiation therapists)
- Personal service workers (e.g. dental hygienists, Aboriginal & Torres Strait Islander health workers, massage therapists, nursing support, personal care workers)
- Management, Administration and Corporate support (e.g. executive, managerial, clerical, reception, finance/ accounting, HR/ payroll, engineer, IT, legal, public relations, health information)
- Support services (e.g. environmental services, food services, cleaning, laundry, gardens/ grounds, building/ equipment maintenance, vehicles/ transport, security, workshop)
- Other (please specify) ____________

Is your primary work role in one of the following areas?

- Aged care
- Critical care
- Drug and alcohol
- Emergency
- Maternity care
- Medical
- Mental health
- Mixed medical/surgical
- Neonatal care
- Palliative care
- Paediatrics
- Peri-operative
- Rehabilitation
- Surgical
- Other (please specify) ____________

Are you the manager of one or more employees?

- Yes

- [ ] No

How many years have you worked in the community health sector?

[ ]

How many years have you been employed at your current organisation?

[ ]

Which of the following community health services do you currently work for?

- [ ] Connect Health
- [ ] Primary Care Connect
- [ ] Ballarat CHS
- [ ] Star Health
- [ ] Gateway Health
- [ ] IPC Health
- [ ] Merri Health
- [ ] cohealth
- [ ] Other (please specify)

Where is your main workplace located?

- [ ] Melbourne CBD
- [ ] Melbourne Suburbs
- [ ] Ballarat
- [ ] Bendigo
- [ ] Geelong
- [ ] Horsham
- [ ] Latrobe (incorporates Traralgon, Morwell, Churchill, Moe)
- [ ] Mildura
- [ ] Shepparton
- [ ] Wangaratta
- [ ] Warrnambool

- ☐ Wodonga
- ☐ Other city or town (please specify)
- ☐ Outside Victoria

Health and well-being

Section 2. Your health and well-being

In general, would you say your health is...?

- ○ Very poor
- ○ Poor
- ○ Fair
- ○ Good
- ○ Excellent

Please read each statement and select the option which indicates how much the statement applied to you *over the past week*. There are no right or wrong answers. Do not spend too much time on any statement.

	Did not apply to me at all	Applied to me to some degree, or some of the time	Applied to me to a considerable degree, or a good part of time	Applied to me very much, or most of the time
I found myself getting upset by quite trivial things	○	○	○	○
I was aware of dryness of my mouth	○	○	○	○
I couldn't seem to experience any positive feeling at all	○	○	○	○
I experienced breathing difficulty (eg, excessively rapid breathing, breathlessness in the absence of physical exertion)	○	○	○	○

	Did not apply to me at all	Applied to me to some degree, or some of the time	Applied to me to a considerable degree, or a good part of time	Applied to me very much, or most of the time
I just couldn't seem to get going	○	○	○	○
I tended to over-react to situations	○	○	○	○
I had a feeling of shakiness (eg, legs going to give way)	○	○	○	○
I found it difficult to relax	○	○	○	○
I found myself in situations that made me so anxious I was most relieved when they ended	○	○	○	○
I felt that I had nothing to look forward to	○	○	○	○
I found myself getting upset rather easily	○	○	○	○
I felt that I was using a lot of nervous energy	○	○	○	○
I felt sad and depressed	○	○	○	○
I found myself getting impatient when I was delayed in any way (eg, lifts, traffic lights, being kept waiting)	○	○	○	○
I had a feeling of faintness	○	○	○	○
I felt that I had lost interest in just about everything	○	○	○	○
I felt I wasn't worth much as a person	○	○	○	○
I felt that I was rather touchy	○	○	○	○
I perspired noticeably (eg, hands sweaty) in the absence of high temperatures or physical exertion	○	○	○	○

	Did not apply to me at all	Applied to me to some degree, or some of the time	Applied to me to a considerable degree, or a good part of time	Applied to me very much, or most of the time
I felt scared without any good reason	○	○	○	○
I felt that life wasn't worthwhile	○	○	○	○

Please indicate the extent to which you agree with each of the following statements:

	Strongly disagree	Disagree	Neutral	Agree	Strongly agree
I tend to bounce back quickly after hard times	○	○	○	○	○
I have a hard time making it through stressful events	○	○	○	○	○
It does not take me long to recover from a stressful event	○	○	○	○	○
It is hard for me to snap back when something bad happens	○	○	○	○	○
I usually come through difficult times with little trouble	○	○	○	○	○
I tend to take a long time to get over set-backs in my life	○	○	○	○	○

Coronavirus concerns and impact

Section 3. The impact of COVID-19 (coronavirus) on you and your work

What is your COVID 19 contact status?

○ No direct contact with people (at work or outside of work) with known COVID 19 diagnosis.

○ Direct contact with people (at work or outside of work) who have had COVID 19 diagnosis which resulted in self-isolation or testing (with a negative COVID 19 result)

○ Diagnosed with COVID 19

During Wave 2 of the COVID-19 pandemic (ie June - November 2020), how concerned were you about...

	Not concerned	Slightly concerned	Somewhat concerned	Very concerned	Extremely concerned	Not applica
Falling ill as a result of COVID-19	○	○	○	○	○	○
Passing COVID-19 on to family members	○	○	○	○	○	○
Your family's health (ie that members of your family may become infected)	○	○	○	○	○	○
Your colleagues having COVID-19	○	○	○	○	○	○
Clients having COVID-19	○	○	○	○	○	○
Caring for a client who has or has suspected COVID-19	○	○	○	○	○	○
Other (please specify) ________	○	○	○	○	○	○

To what extent do you agree or disagree with each of the following statements about the impact of COVID-19 (coronavirus) in general on you at work?

	Strongly disagree	Disagree	Neither disagree nor agree	Agree	Strongly agree	Prefer not to answer / Don't know/Not applicable
My job puts me at risk of getting COVID-19	○	○	○	○	○	○
I feel more stress at work	○	○	○	○	○	○
I have had to do work tasks that I don't usually do	○	○	○	○	○	○
I was redeployed to another role	○	○	○	○	○	○
I have had to do more work than I usually do	○	○	○	○	○	○
I had to significantly change the way I delivered service to my clients/stakeholders	○	○	○	○	○	○

	Strongly disagree	Disagree	Neither disagree nor agree	Agree	Strongly agree	Prefer not to answer / Don't know/Not applicable
I worked from home/remotely	○	○	○	○	○	○
I provided services to some/all of my clients/stakeholders via telehealth	○	○	○	○	○	○
The situation has brought me closer to my manager	○	○	○	○	○	○
There is more conflict amongst colleagues at work	○	○	○	○	○	○
There is an increased sense of togetherness and cooperation among the staff	○	○	○	○	○	○
I have considered resigning because of COVID-19	○	○	○	○	○	○
I have considered taking early retirement because of COVID-19	○	○	○	○	○	○
I have considered transitioning into other sectors because of COVID-19	○	○	○	○	○	○
My awareness and knowledge of disease control has increased	○	○	○	○	○	○
It has been a learning experience	○	○	○	○	○	○
I have been less busy than usual	○	○	○	○	○	○
I have had to cancel or postpone my annual leave because of the COVID-19 outbreak	○	○	○	○	○	○
I am disappointed that I have had to cancel or postpone my annual leave due to COVID-19	○	○	○	○	○	○
I have had to retrain or do training courses so I can do a role/job I normally wouldn't	○	○	○	○	○	○
I don't feel very prepared to care for clients with COVID-19	○	○	○	○	○	○

	Strongly disagree	Disagree	Neither disagree nor agree	Agree	Strongly agree	Prefer not to answer / Don't know/Not applicable
Other, please specify ☐	○	○	○	○	○	○

To what extent do you agree or disagree with each of the following statements about the impact of COVID-19 (coronavirus) in general on you outside of work?

	Strongly disagree	Disagree	Neither disagree nor agree	Agree	Strongly agree	Prefer not to answer / Don't know / Not applicable
People close to me have been concerned about my health	○	○	○	○	○	○
The COVID-19 situation has brought me closer to my family	○	○	○	○	○	○
People treat me and my family differently because I work in the community health sector	○	○	○	○	○	○
People avoid me and my family because I work in a community health service	○	○	○	○	○	○
I avoid telling people that I work in the community health sector	○	○	○	○	○	○
I am likely to suffer financial losses	○	○	○	○	○	○
My personal or family's lifestyle has been affected	○	○	○	○	○	○
I have a greater appreciation of life and work	○	○	○	○	○	○
I have avoided public or crowded spaces (eg shops, public transport)	○	○	○	○	○	○
I have avoided interacting with my friends and extended family	○	○	○	○	○	○

	Strongly disagree	Disagree	Neither disagree nor agree	Agree	Strongly agree	Prefer not to answer / Don't know / Not applicable
My family and friends are worried they might get infected from me	○	○	○	○	○	○
Other (please specify)	○	○	○	○	○	○

My organisation's response

Section 4. My organisation's response to COVID-19 (coronavirus)

With respect to Wave 2 of the COVID-19 pandemic (June - November 2020), how would you rate your organisation's ...

	Very poor	Poor	Neither poor nor good	Good	Excellent
Level of preparedness?	○	○	○	○	○
Availability and use of precautionary measures eg PPE such as masks?	○	○	○	○	○
Communication with staff?	○	○	○	○	○
Training provided to staff, eg in use of masks?	○	○	○	○	○
Concern for the physical well-being of staff?	○	○	○	○	○
Concern for the emotional well-being of staff?	○	○	○	○	○
Availability of emotional support for those who needed it?	○	○	○	○	○

For each of the following precautionary measures, please indicate to what extent they interfere or interfered with your work

	Does not affect my ability to do my job	Affects my ability to do my job a little	Affects my ability to do my job a lot	Not applicable
Mask	○	○	○	○
Face shields	○	○	○	○
Gloves	○	○	○	○
Goggles / eye shields	○	○	○	○
More frequent hand-washing or sanitising	○	○	○	○
Restricted access to some or all sites	○	○	○	○
Restricted face-to-face meetings or gatherings	○	○	○	○
Physical ('social') distancing from colleagues	○	○	○	○
Physical ('social') distancing from clients	○	○	○	○
Staying away from work when you have any signs of illness	○	○	○	○
Other, please specify ________	○	○	○	○

Open-ended

Section 5. Any other comments

Have we missed anything? If you have anything else you would like to tell us about the impact of COVID-19 on you or your role or your organisation's response, please write in the box below.

____

Block 6

Everyone who completes the survey is eligible to go into the draw to win one of two **$100 gift vouchers**.

We would also like to send you a **summary of the findings** once they are available, if you are interested.

Would you like to participate in the draw and/or receive a summary of the findings?

- Yes
- No

Block 7

Thank you for completing this questionnaire.

Your responses will contribute to understanding the impact and psychosocial needs of community health service staff in situations such as COVID-19, and inform your organisation's response to future outbreaks of infectious diseases.

Deakin University CRICOS Provider Code 00113B.

Powered by Qualtrics
